# Trypanocidal activity of salinomycin is due to sodium influx followed by cell swelling

**DOI:** 10.1186/1756-3305-6-78

**Published:** 2013-03-21

**Authors:** Dietmar Steverding, Darren W Sexton

**Affiliations:** 1 BioMedical Research Centre, Norwich Medical School, University of East Anglia, Norwich Research Park, Norwich, NR4 7TJ, UK

**Keywords:** African trypanosomiasis, *Trypanosoma brucei*, Salinomycin, Drug screening

## Abstract

**Background:**

The few currently available drugs for treatment of African trypanosomiasis are outdated and show problems with toxicity and resistance. Hence, there is an urgent need for the discovery and development of new anti-trypanosomal agents.

**Findings:**

In this study, the ionophorous antibiotic salinomycin was investigated for its trypanocidal activity *in vitro* using culture-adapted bloodstream forms of *Trypanosoma brucei*. The concentrations of salinomycin to reduce the growth rate by 50% and to kill the parasites were 0.31 μM and 1 μM, respectively. The trypanocidal action of the ionophore was shown to be the result of an influx of Na^+^ resulting in an increased intracellular Na^+^ concentration followed by cell swelling. This mode of action differs from the mechanism for the anti-cancer activity of salinomycin reported to be by induction of apoptosis.

**Conclusion:**

Here we have shown that salinomycin is an effective agent against bloodstream forms of *T. brucei* and might be a potential candidate for treatment of African trypanosomiasis.

## Findings

### Background

African trypanosomes are the etiological agents of sleeping sickness in humans and nagana disease in cattle. The parasites are transmitted by the bite of infected tsetse flies and live and multiply in the blood and tissue fluids of their mammalian hosts. Both sleeping sickness and nagana disease occur in sub-Saharan Africa between 14° North and 20° South latitude, the distribution area of tsetse flies [1]. In this so-called tsetse belt, millions of people and cattle are at risk of getting infected with the parasites [2,3]. In addition, it is estimated that nagana disease costs the affected African countries over 1 billion USD per year [3]. Chemotherapy of African trypanosomiasis still relies on a few drugs developed decades ago, most of which show poor efficacy and significant toxicity, and are being increasingly subject to drug resistance [4]. Thus, new drugs are urgently needed for chemotherapy of sleeping sickness and nagana disease. One approach for the discovery of new drugs for treatment of African trypanosomiasis is the screening of existing drugs for trypanocidal activities [5].

**Figure 1:**
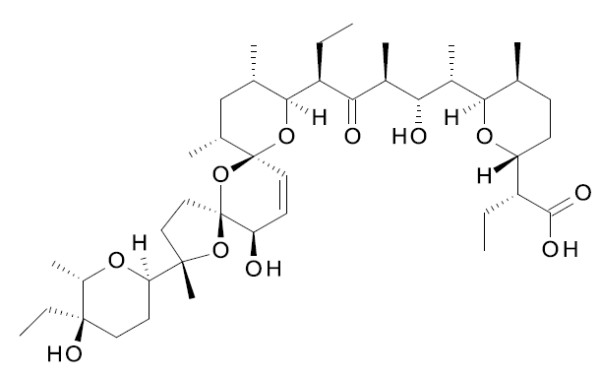
Structure of salinomycin.

Salinomycin (Figure 1) is a carboxylic polyether antibiotic produced by a strain of *Streptomyces albus*[6]. It is a monovalent cation ionophore mediating the transport of Na^+^, K^+^ and Rb^+^ (but not of Cs^+^, Mg^2+^, Ca^2+^ and Sr^2+^) across organic phases [7]. The antibiotic is widely used as a food supplement to control coccidiosis in poultry [8,9] and has recently received attention as a novel cancer drug candidate [10]. However, compounds displaying anti-cancer activity usually also exhibit strong trypanocidal activity [11-13]. In this study, we investigated the trypanocidal activity of salinomycin and the underlying mechanism of action of the antibiotic using bloodstream forms of *Trypanosoma brucei*.

### Methods

The trypanocidal activity of salinomycin was determined with bloodstream forms of the *T. brucei* clone 427–221 [14] while the general cytotoxicity of the ionophore was evaluated with human HL-60 cells and normal PBMCs (peripheral blood mononuclear cells). Cells were seeded in 24-well plates in a final volume of 1 ml of appropriate culture medium (trypanosomes: Baltz medium [15]; human cells: RPMI medium [16]) supplemented with 16.7% (v/v) heat-inactivated foetal bovine serum and containing various concentrations of salinomycin (10^-4^ to 10^-9^ M) and 1% DMSO. Wells containing medium and 1% DMSO served as controls. The initial cell densities were 1 × 10^4^/ml for trypanosomes, 1 × 10^5^/ml for HL-60 cells and 5 × 10^5^/ml for PBMCs. After 24 h incubation, 100 μl of a 0.44 mM resazurin solution prepared in PBS was added and the cells were incubated for a further 48 h. Thereafter, the plates were read on a microplate reader using a test wavelength of 570 nm and a reference wavelength of 630 nm. The 50% growth inhibition (GI_50_) value (trypanosomes and HL-60 cells) and the 50% effective concentration (EC_50_) value (PBMCs), i.e., the concentration of salinomycin necessary to reduce the growth rate of cells by 50% or to cause an effect in 50% of cells compared to the control, were determined from mean values using the 4-parameter logistic model [17]. The following formula for the 4-parameter logistic model was used: *Y* = {[*a*-*d*]/[1 + (*X*/*c*)^*b*^]} + *d*, where *Y* is the response, *X* is the concentration, *a* is the lower asymptote (lower plateau), *d* is the upper asymptote (upper plateau), *b* is the slope factor (Hill factor) and *c* is the GI_50_/EC_50_ value. The minimum inhibitory concentration (MIC) value, i.e. the concentration of salinomycin at which all cells were killed, was determined microscopically by inspecting each well thoroughly for the presence of any motile trypanosomes or unlysed HL-60s or PBMCs.

Changes in cell volume were measured using a previously described light scattering method [18]. Bloodstream forms of *T. brucei* were seeded at a density of 5 × 10^7^ cells/ml in 96-well plates in a final volume of 200 μl culture medium containing 100 μM salinomycin and 0.5% DMSO (test) or 0.5% DMSO alone (control). Absorbance of the cultures was measured at 490 nm every 15 min. A decrease in absorbance corresponded to an increase in cell volume.

Cell morphology changes were examined by light microscopy. Briefly, bloodstream forms of *T. brucei* were treated at a density of 5 × 10^7^ cells/ml with 100 μM salinomycin and 0.5% DMSO (test) or 0.5% DMSO alone (control). After 1 h incubation, cells were fixed with 2% formaldehyde/0.05% glutaraldehyde in PBS, spread onto slides and air dried. The smears were stained with May-Grünwald staining solution and then imaged with a Zeiss Axioplan 2 fluorescence microscope using a Plan-Apochromat 100×/1.4 oil objective.

The intracellular level of Na^+^ was monitored with Sodium Green™ tetraacetate, a cell-permeant indicator for the fluorometric determination of Na^+^ concentration. The probe can freely diffuse across cell membranes and is intracellularly converted into the Na^+^-responsive acidic form by esterases. Trypanosomes were incubated at a density of 5 × 10^7^ cells/ml with 100 μM salinomycin and 0.5% DMSO (test) or 0.5% DMSO alone (control) in the presence of 4 μM Sodium Green™ tetraacetate for 1 h. After washing twice with culture medium, cells were then immediately analysed by flow cytometry using a BD Accuri C6 flow cytometer. The excitation wavelength was 488 nm and the filter set was 530/30 nm. Gates were set to exclude cell fragments and debris from the analysis, and 50,000 gated cells were analysed.

### Results and discussion

**Figure 2:**
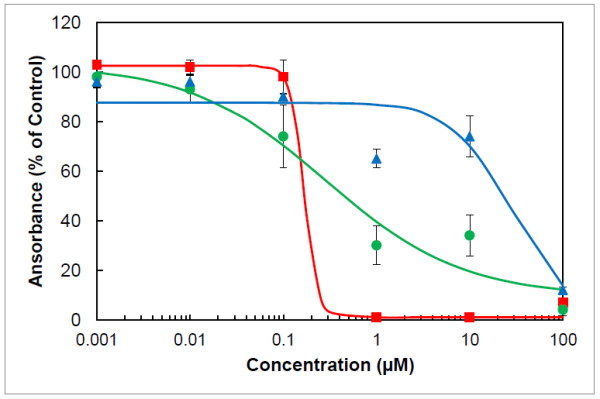
**Trypanocidal and cytotoxic effects of salinomycin.** Bloodstream forms of *T. brucei* (red squares), HL-60 cells (green circles) and PBMCs (blue triangles) were incubated with varying concentrations of salinomycin. After 72 h of culture, cell viability and proliferation were determined with the colorimetric dye resazurin. Mean values ± SD of three (PBMCs) or four (*T. brucei* and HL-60) experiments are shown. Dose–response curves were calculated from mean values using the 4-parameter logistic model.

Salinomycin showed a dose-dependent effect on the growth of *T. brucei* bloodstream forms with a GI_50_ value of 0.17 μM and a MIC value of 1 μM (Figure 2). The ionophore displayed similar cytotoxic activity against leukaemic HL-60 cells with a GI_50_ value of 0.29 μM and a MIC value of 1 μM (Figure 2). Similar antiproliferative activity has been recently reported for various other cancer cells including human promyelocytic leukaemia cells (IC_50_ = 0.44 μM [19]), human colon adenocarcinoma cells (IC_50_ = 1.11 μM [19]) and VCaP prostate carcinoma cells (EC_50_ = 0.38 μM [20]). In contrast to the effect on cancer cells, it was reported that salinomycin is 10–100 times less cytotoxic against non-malignant cells [19,20]. In fact, salinomycin exhibited only low cytotoxicity against normal PBMCs with an EC_50_ value of 29.9 μM and a MIC value of 100 μM (Figure 2). This latter finding indicates that the ratio for cytotoxic/trypanocidal activity (selectivity index) for salinomycin is in a moderate range.

One obvious mechanism of action of salinomycin would be its effect as an ionophore on the depolarisation of membrane potentials. By using the fluorescent probe 3,3’-dihexyloxycarbocyanine, however, it was found that salinomycin did not affect the membrane potential of bloodstream forms of *T. brucei* (data not shown). This observation is in agreement with previous findings that valinomycin also did not influence the membrane potential of trypanosomes, although the parasites are extremely sensitive to this ionophore [21].

**Figure 3:**
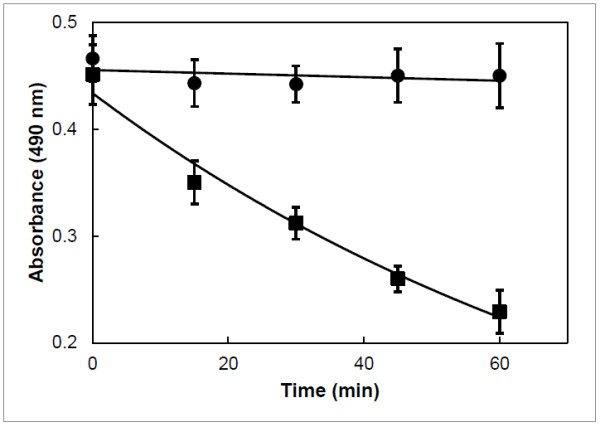
**Effect of salinomycin on the cell volume of bloodstream forms of *T. brucei*.** Trypanosomes (5 × 10^7^ cell/ml) were incubated in the absence (circles) or presence (squares) of 100 μM salinomycin in culture medium. Every 15 min the absorbance at 490 nm was measured. Note that a decrease in absorbance corresponds to an increase in cell volume. Mean values ± SD of three experiments are shown.

**Figure 4:**
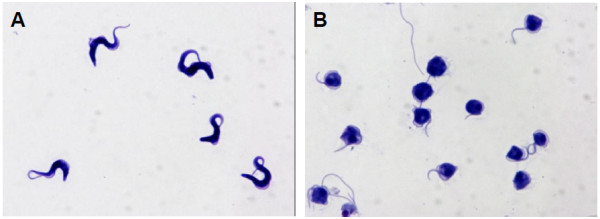
**Effect of salinomycin on the morphology of bloodstream forms of *T. brucei*.** Trypanosomes (5 × 10^7^ cell/ml) were incubated in the absence (**A**) or presence (**B**) of 100 μM salinomycin for 1 h. Formaldehyde/glutaraldehyde-fixed cells were spread onto slides, air dried and stained with May-Grünwald staining solution. A representative experiment out of three is shown.

**Figure 5:**
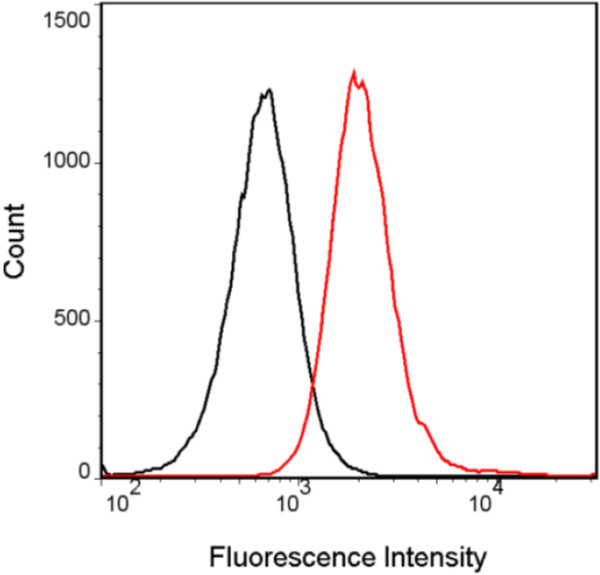
**Effect of salinomycin on intracellular Na**^**+ **^**levels in bloodstream forms of *T. brucei*.** Trypanosomes (5 × 10^7^ cell/ml) were incubated with 4 μM Sodium Green™ tetraacetate in the absence (black line) or presence (red line) of 100 μM salinomycin. After 1 h incubation, trypanosomes were washed with culture medium and analysed by flow cytometry. A representative experiment out of three is shown.

Another possible mechanism of action of salinomycin could be that the ionophore increases the intracellular cation level followed by a quick entry of water which would lead to cell swelling and damage of intracellular structures. Changes in cell volume can be monitored spectrophotometrically by following the absorbance of the cell suspension at 490 nm. Incubation of bloodstream forms of *T. brucei* with 100 μM salinomycin resulted in a decrease of absorbance over time while the absorbance of control cultures did not change (Figure 3). It should be noted that a much higher concentration of salinomycin was needed (100 μM compared to 1 μM necessary to kill all cells in the growth inhibition assay shown in Figure 2) in order to observe a measurable effect on the swelling of trypanosomes in a short period of time. This is because that at high cell density, which is required for the recording of changes in cell volume, bloodstream forms of *T. brucei* do not survive for a very long time in culture. The decrease in absorbance corresponded to cell swelling as judged from visual observation by light microscopy. Parasites treated for 1 h with 100 μM salinomycin lost the normal elongated shape of trypanosomes and appeared as rounded up cells (Figure 4). The only monovalent cations present within bloodstream forms of *T. brucei* and in the culture medium are Na^+^ and K^+^. Thus, only these may be involved in the cell swelling observed in the presence of salinomycin. As bloodstream forms of *T. brucei* are low-Na^+^ and high-K^+^ cells [22], only a salinomycin-mediated transport of Na^+^ from the exterior to the interior could explain the observed cell swelling. To prove that the cause of the swelling was indeed due to an increased intracellular Na^+^ level, the concentration of Na^+^ was determined with the fluorescent probe Sodium Green™ tetraacetate. Subsequent flow cytometry revealed that trypanosomes treated with 100 μM salinomycin for 1 h had a much higher fluorescence signal than control cells (Figure 5). The median fluorescence intensity signal for salinomycin-treated parasites was 2223 ± 164 (n = 3) and for control parasites it was 1050 ± 345 (n = 3). This result indicates that upon incubation with salinomycin, the intracellular Na^+^ concentration was raised more than twofold in the trypanosomes from 13.7 mM [22] to about 30 mM. This increase in Na^+^ concentration is enough to explain the swelling of trypanosomes by entry of water. For instance, subjection of bloodstream forms of *T. brucei* to a reduction in osmolarity from 300 to 150 mOsm (which in turn can be regarded as a twofold increase in the intracellular concentration of solute particles) results in a dramatic initial swelling of the cells [23].

### Conclusion

This study has demonstrated that the ionophore salinomycin displays trypanocidal activity by a mechanism involving increased Na^+^ influx followed by subsequent cell swelling which is probably due to the uptake of water. This mechanism is different from the mode of action reported for the anti-cancer activity of salinomycin. Recent research has shown that salinomycin induces apoptosis in cancer cells by different mechanisms, which include increasing the intracellular levels of reactive oxygen species (ROS) [24] and inhibiting the Wnt signalling pathway [25]. However, we did not find any evidence that the trypanocidal activity of salinomycin is associated with the induction of apoptosis in bloodstream forms of *T. brucei* (data not shown).

Importantly, salinomycin can be administered orally whereas most of the current drugs used for treatment of African trypanosomiasis have to be given parenterally. Before developing salinomycin into an anti-trypanosomal agent, animal experiments are needed to establish the *in vivo* trypanocidal activity of the ionophore. However, a selectivity index of 10–100 may be inadequate for proceeding with animal studies as it is recommended that such animal experiments should only be pursued if the selectivity index is greater than 100 [26]. Despite this, salinomycin may serve as a lead for the development of derivatives with improved trypanocidal activities. In addition, the ionophore could be used in combination with current anti-trypanosomal drugs. In recent years, such a drug combination regime (eflornithine/nifurtimox) has been successfully introduced for the treatment of human African sleeping sickness [27].

## Competing interests

The authors have no competing interests.

## Authors’ contribution

DS designed the experiments. DS and DWS performed the experiments. DS drafted the manuscript. DWS edited the manuscript. Both authors read and approved the final manuscript.
